# Multimodal Ultrasound Radiomic Technology for Diagnosing Benign and Malignant Thyroid Nodules of Ti-Rads 4-5: A Multicenter Study

**DOI:** 10.3390/s24196203

**Published:** 2024-09-25

**Authors:** Luyao Wang, Chengjie Wang, Xuefei Deng, Yan Li, Wang Zhou, Yilv Huang, Xuan Chu, Tengfei Wang, Hai Li, Yongchao Chen

**Affiliations:** 1 Department of Ultrasound, The First Affiliated Hospital of Anhui Medical University, Hefei 230022, China; 2245011713@stu.ahmu.edu.cn (L.W.); lee3647254@163.com (Y.L.); zhouwangayfycsk@163.com (W.Z.); 2345011871@stu.ahmu.edu.cn (Y.H.); 2 Anhui Medical University, Hefei 230032, China; 2245011272@stu.ahmu.edu.cn; 3 School of Basic Medical Sciences, Anhui Medical University, Hefei 230032, China; dengxf@ahmu.edu.cn; 4 Hefei Cancer Hospital, Chinese Academy of Sciences, Hefei 230031, China; chuxuan@cmpt.ac.cn; 5 Anhui Province Key Laboratory of Medical Physics and Technology, Institute of Health and Medical Technology, Hefei Institutes of Physical Science, Chinese Academy of Sciences, Hefei 230031, China

**Keywords:** radiomics, thyroid nodules, multimodal ultrasound, elastography

## Abstract

This study included 468 patients and aimed to use multimodal ultrasound radiomic technology to predict the malignancy of TI-RADS 4-5 thyroid nodules. First, radiomic features are extracted from conventional two-dimensional ultrasound (transverse ultrasound and longitudinal ultrasound), strain elastography (SE), and shear-wave-imaging (SWE) images. Next, the least absolute shrinkage and selection operator (LASSO) is used to screen out features related to malignant tumors. Finally, a support vector machine (SVM) is used to predict the malignancy of thyroid nodules. The Shapley additive explanation (SHAP) method was used to intuitively analyze the specific contributions of radiomic features to the model’s prediction. Our proposed model has AUCs of 0.971 and 0.856 in the training and testing sets, respectively. Our proposed model has a higher prediction accuracy compared to those of models with other modal combinations. In the external validation set, the AUC of the model is 0.779, which proves that the model has good generalization ability. Moreover, SHAP analysis was used to examine the overall impacts of various radiomic features on model predictions and local explanations for individual patient evaluations. Our proposed multimodal ultrasound radiomic model can effectively integrate different data collected using multiple ultrasound sensors and has good diagnostic performance for TI-RADS 4-5 thyroid nodules.

## 1. Introduction

Thyroid cancer is a widely distributed cancer worldwide [1]. With the advancement of modern medical technology, the detection rate of thyroid cancer has gradually increased in recent years [2]. Currently, fine-needle aspiration biopsy (US-FNAB) is a reliable, commonly used, and widely accepted method for diagnosing benign and malignant thyroid nodules [3]. However, this is an invasive examination method [4,5], and its accuracy and practicality depend, to some extent, on the operator’s proficiency and personal experience [6]. In addition, some pathological results of thyroid nodules are suspicious or uncertain, which may require patients to undergo another puncture or undergo a diagnostic thyroidectomy [6], thereby increasing the patient’s risk (such as for permanent parathyroid dysfunction and recurrent laryngeal nerve injury) and increasing the patient’s economic burden. Therefore, improving the accuracy for diagnosing thyroid malignant nodules is crucial.

Conventional ultrasound (US) is the preferred imaging method for screening thyroid nodules [6,7]. However, studies have shown that the accuracy of US in distinguishing between benign and malignant nodules is relatively low [8]. At present, thyroid nodules in US images are widely classified for malignancy using the Thyroid-Imaging Radiology and Data System (TI-RADS) developed by the American Society of Radiology (ACR) [9,10,11]. Although this standard is widely adopted, its highest accuracy is only 59.93% [12].

Ultrasound elastography (USE) is a new technology that reflects and measures tissue hardness based on the principle that diseased tissue is usually harder than benign or normal tissue [13]. According to the applied external force, USE can be divided into strain elastography (SE) and shear wave elastography (SWE) [14,15]. SE evaluates tissue elasticity by compressing tissue displacement, while SWE evaluates tissue elasticity by measuring the propagation speed of transverse waves [16,17]. Many studies have demonstrated the effectiveness of USE in distinguishing between benign and malignant thyroid nodules [18]. In addition, the combination of US and USE has been proven to improve recognition ability significantly [19,20,21]. However, the interpretation of ultrasound images in clinical practice often relies on the experience of radiologists, which has particular subjectivity and limitations.

To address these issues, there is an urgent need for a convenient and non-invasive ultrasound-assisted diagnostic tool in clinical practice to help radiologists accurately determine thyroid nodules’ benign or malignant nature, thereby reducing unnecessary biopsies. Radiomics is a new field of medical image research, which further expands the quantitative analysis of medical images [22]. Unlike doctors using machine vision to observe lesions, radiomics converts medical images to high-throughput quantitative features through computer algorithms [23]. Ultrasound radiomics is a branch of radiomics that extracts and analyzes quantitative imaging features from ultrasound images and can obtain features such as tumor shapes, textures, and wavelets, providing valuable diagnostic, prognostic, or predictive information for clinical doctors [24]. Ultrasound radiomics has shown good ability in detecting and classifying thyroid nodules in ultrasound images [25,26,27]. On this basis, researchers have combined multimodal ultrasound images with radiomics to further improve the classification performance of thyroid nodules. For example, Chen et al. applied radiomics to extract radiomic features from routine thyroid and contrast-enhanced ultrasound images and classified and predicted benign and malignant thyroid nodules [28]. Wang et al. established a multimodal ultrasound radiological joint diagnostic model by analyzing US and SWE images [29]. Zhang et al. used radiomics to interpret ultrasound images of SE and SWE and developed a nomogram that combines radiomic scores from multimodal ultrasound images with independent clinical features to predict malignant thyroid nodules [30]. The above studies indicate ultrasound radiomics can effectively distinguish between benign and malignant thyroid nodules through ultrasound image features.

However, the above research has certain limitations and challenges. First, USE and contrast-enhanced ultrasound have not been widely used in clinical practice, and there are limited data available for modeling, which can lead to overfitting. Second, because of institutional bias and protocol changes, single-center studies lack generalizability, and multicenter validation is crucial for reliable radiomic models. Finally, the decision-making of radiomic models is opaque and difficult to understand, and it is necessary to combine clinical features to make radiologists understand and trust them.

This study used four modes of ultrasound images, including two types of USE (both SE and SWE) and two types of US (transverse US and longitudinal US), to provide more prosperous and complete information for diagnosing benign and malignant thyroid nodules through radiomic analysis methods. Meanwhile, we validated the effectiveness and stability of the proposed model at multiple centers. Finally, we combined the model with the SHAP method to explain and visualize the decision-making process of the proposed model, thereby improving its interpretability and clinical practicality.

This study integrated data from the same sensor in different modes, covering two types of ultrasound elastography (SE and SWE) and two types of ultrasound scans (transverse US and longitudinal US). Through these data, we have provided richer and more comprehensive information for diagnosing benign and malignant thyroid nodules using radiological analysis methods. This method is applicable to single-modal data collected from sensors and can fully utilize the potential of multimodal data, significantly improving the diagnostic performance and the broad applicability of the model. In addition, we validated the proposed model at multiple medical centers to ensure its effectiveness and stability. To further enhance the interpretability and clinical practicality of the model, we plan to combine it with the SHAP (Shapley additive explanation) method. Through this combination, we will be able to explain and visualize the decision-making process of the model, making its application more intuitive and understandable and enhancing its value in clinical practice.

## 2. Materials and Methods

### 2.1. Study Population 

We retrospectively collected the ultrasound images of 508 cases of thyroid nodules with thyroid FNAB, from March 2023 to December 2023 at the First Affiliated Hospital of Anhui Medical University (Jixi Road Campus and Gaoxin Campus) and Hefei Cancer Hospital of the Chinese Academy of Sciences, as research materials, using Mindray Resona 7S, Resona 7EXP, Eagus R9T, and color Doppler ultrasound diagnostic equipment and a linear array high-frequency probe, L14-5WU (at frequencies in the range 5–14 Hz), for examination. All the machines are equipped with elastic imaging software. All the benign and malignant results of the nodules are based on pathological biopsy results as the gold standard.

**Figure 1:**
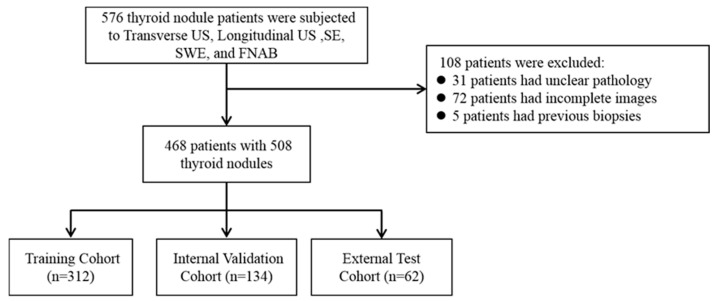
The inclusion process of the research population.

Our inclusion criteria are as follows: (1) All the patients must undergo complete routine two-dimensional ultrasound (transverse US and longitudinal US), ultrasound strain elastography (SE), and ultrasound shear wave (SWE) examinations by ultrasound physicians with at least five years of work experience and standardized training; (2) All the modal images collected are complete and of good quality; (3) All the patients underwent FNAB or surgical treatment (a total or subtotal thyroidectomy) under ultrasound guidance; (4) All the lesions were not treated or treated before the ultrasound examination (biopsy, thermal ablation, or thyroidectomy). The exclusion criteria are as follows: (1) Patients who have previously undergone a biopsy, thyroid surgery, or thermal ablation treatment; (2) those whose histological or cytopathological results are unable to diagnose or whose diagnosis is unclear; (3) poor quality ultrasound or elastography images. This study was approved by all the centers’ ethics committees (2023-516 and PJ-KY2023-113), and all the studies complied with relevant regulations. The inclusion process of the study population is shown in Figure 1. According to the above criteria, the data of the three centers participating in this study are as follows:

Cohort 1: The model training and internal testing included 312 patients from the Jixi Road Campus of the First Affiliated Hospital of Anhui Medical University;

Cohort 2: A total of 134 patients from the Gaoxin Campus of the First Affiliated Hospital of Anhui Medical University were included in model training and internal testing;

Cohort 3: A total of 62 patients from Hefei Cancer Hospital, Chinese Academy of Sciences, were included for external validation of the model.

### 2.2. Image Acquisition and Segmentation

Ultrasound physicians with over five years of work experience perform standardized thyroid nodular scans using US, SE, and SWE. First, a routine ultrasound examination is performed, with the patient in a supine position, breathing calmly, and the probe perpendicular to the patient’s neck. The thyroid nodule’s optimal transverse and longitudinal views (i.e., the maximum diameter section) are obtained. At the same time, the malignant features of thyroid nodules are recorded using US (boundary, aspect ratio, composition, echo, maximum diameter, presence or absence of calcification, and blood supply status) and classified according to ACR-TIRADS. Subsequently, the imaging is switched to the SE mode, and the patient is instructed to hold their breath. When the image is stable (the pressure indicator’s green bar is stable near the indicator line), the best elastic image is obtained, and the strain values (SE means) of the diseased tissue and normal tissue are obtained at the same level. The instrument automatically displays the nodule’s strain ratio (SR) and takes the average of three measurements. Finally, imaging is switched to the SWE mode for the same patient and nodule; the patient is instructed to hold their breath while waiting for the image to stabilize (M-STB index rating ≥ four stars) to obtain the best image. The maximum and average Young’s modulus values (Emax and Emean) of the nodule are measured three times to obtain the average.

Two ultrasound physicians, who have worked in thyroid disease diagnosis for more than five years, manually drew regions of interest (ROIs) on nodules in multimodal ultrasound images using ITK-SNAP3.8.0 software.

### 2.3. Radiomic Feature Extraction and Selection

Radiomic features were automatically extracted from multimodal images of each target nodule using the Pyradiomics toolkit [31], including histogram features, features based on the gray-level co-occurrence matrix (GLCM), features based on the gray-level run length matrix (GLRLM), features based on the gray-level size zone matrix (GLSZM), and wavelet features. Among them, SE and SWE have three channel features, so it is necessary to extract basic features from the red (R), green (G), and blue (B) channels in the image. A total of 50 nodules were randomly selected from the training set, and another ultrasound physician, who has been working for more than five years, described them using the same method. The intra-group correlation coefficient (ICC) was calculated for the data obtained by the two physicians to evaluate the repeatability of the radiomic feature acquisition. The results indicate these features have good repeatability (ICC > 0.75). In this study, ICC > 0.75 was adopted as the standard for the feature consistency assessment. This threshold is widely used in medical image analysis to judge the stability and consistency of features in different data sets or repeated experiments. According to Koo and Li et al. (2016) [32], ICC values greater than 0.75 represent good reproducibility and meet the criteria for clinical application, ensuring the model’s reliability when processing different modal and sensor data. In future work, as data quality and quantity improve, our models must maintain this high standard of consistency assessment.

We used redundancy analysis and LASSO to screen features with *p* < 0.05 and ultimately chose 35 variables for the machine-learning model’s construction. 

### 2.4. Model Building and Explanation

We constructed a model for predicting the malignancy of thyroid nodules based on the radiomic features of multimodal ultrasound images. A widely used machine-learning classifier, a support vector machine (SVM), was used for predictions [33]. The hyperparameters in the training set were optimized using the grid search method and 5-fold cross-validation. Finally, the model with the highest area-under-the-curve (AUC) value was selected to verify the testing set’s performance [34].

**Figure 2:**
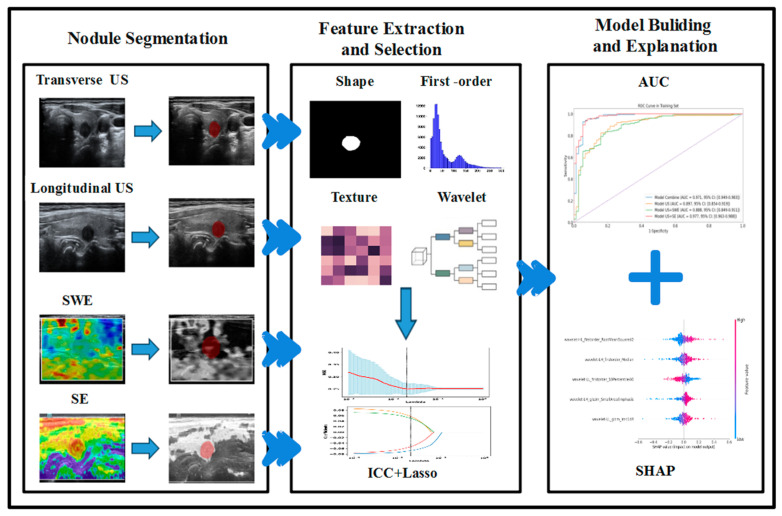
Workflow of radiomic analysis and model building.

Considering the properties of machine-learning black-box models, we use the Shapley additive explanation (SHAP) method to calculate and visualize the impact of each feature on the model’s output to interpret the model’s prediction results [35,36,37]. The overall workflow is shown in Figure 2.

### 2.5. Statistical Analysis

Statistical analysis was conducted for the radiomic characteristics of US, SE, and SWE using SPSS 25.0 and R Studio 4.1.0 software. Continuous variables are represented by (means ± S.D.s) or medians [P25, P75], while categorical variables are represented by frequencies. Independent sample t-tests or rank-sum tests were used to compare the differences between continuous variable groups, and chi-squared tests or Fisher’s exact tests were used to compare the differences between categorical variable groups based on data distribution characteristics. We used the AUC as the primary evaluation metric for the internal testing set and external validation set. In addition, we also calculated the accuracy, sensitivity, and specificity of the model.

## 3. Results

### 3.1. Patient Characteristics

For model training, the nodules in Cohort 1 and Cohort 2 were randomly divided into a training set and an internal testing set, at a ratio of 7:3. Our study ultimately included 508 cases of thyroid nodules (in a total of 468 patients), including 312 in the training set, 134 in the internal testing set, and 62 in the external validation set from Cohort 3. The characteristics of the nodules are shown in Table 1.

### 3.2. Model Evaluation

**Figure 3:**
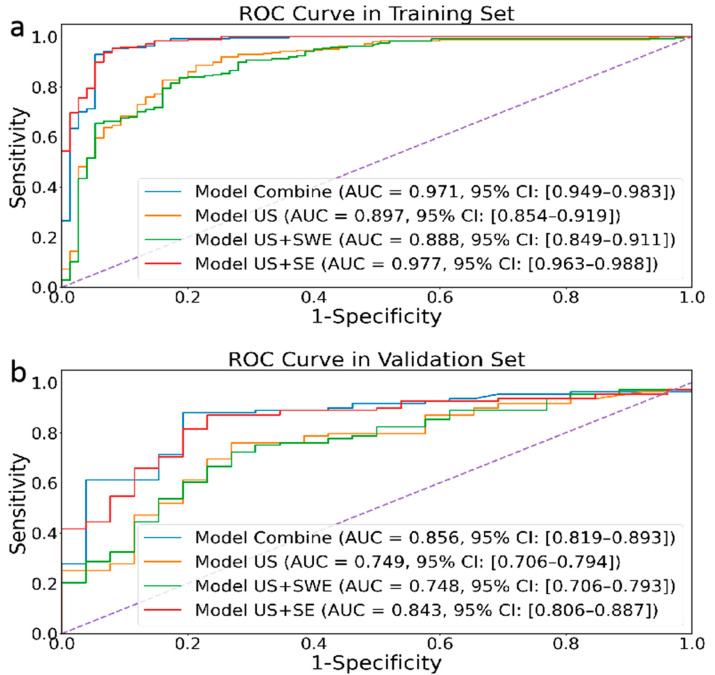
Performances of four different modal image models in the retrospective training (**a**) and validation (**b**) cohorts.

As shown in Figure 3, our proposed radiomic joint model based on multimodal ultrasound image (US (transverse and longitudinal), SE, and SWE) features has AUCs of 0.971 (95% CI: 0.949–0.983) and 0.856 (95% CI: 0.819–0.893) in the training set and internal testing set, respectively. Meanwhile, the joint model’s accuracy, sensitivity, and specificity in the training and validation sets are also higher than those of other groups (Table 2), indicating excellent diagnostic performance.

### 3.3. External Validation of Model Accuracy

Our study divided the data from two hospitals, Cohort 1 and Cohort 2, into a training set and an internal testing set and improved the model’s generalization through data augmentation. We used Cohort 3 hospitals as the external validation set to verify the model’s generalization for other hospitals. The results showed that the AUC of the proposed model was 0.779 (95% CI: 0.716–0.834), with an accuracy of 0.806, a sensitivity of 0.818, and a specificity of 0.714.

### 3.4. The Interpretability of the Model

**Figure 4:**
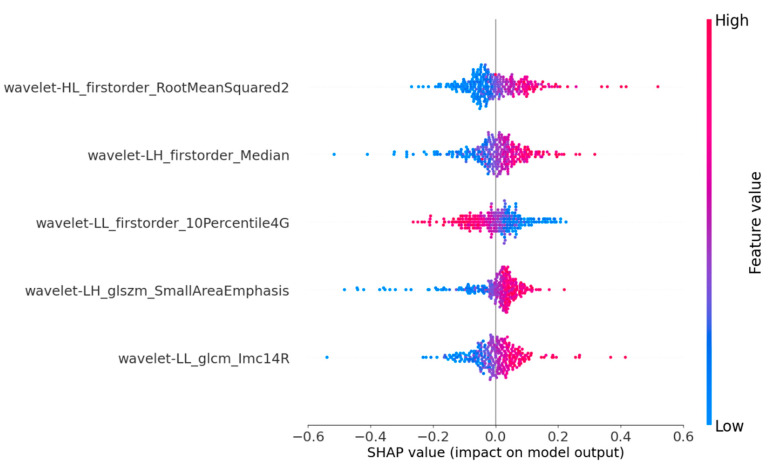
SHAP summary plots of our proposed model. The plot illustrates the feature relevance and combined feature attributions to the model’s predictive performance.

SHAP provides quantitative explanations for radiomic models. This method offers a visually concise graph by displaying the range and distribution of the importance of features to the model’s output and linking feature values with their influences. To gain a deeper understanding of the interpretability of the proposed model, we conducted a thorough analysis of the SHAP values associated with each feature. By displaying SHAP values as a bar chart, the evaluation of individual patients can be intuitively explained, indicating each feature’s positive or negative contributions to the model’s output. Figure 4 shows that the five most important features for predicting nodular malignancy, as determined using SHAP, were processed through the wavelet transform [38,39]. The wavelet transform can effectively extract multiscale and multidirectional information from images, thereby capturing more valuable features that help to distinguish between benign and malignant nodules. The following is a specific analysis of these five essential features:

(1) Feature 1 (Wavelet_HL_firstorder-RootMeanSquared2): By extracting horizontal edge information from longitudinal ultrasound images and describing the pixel intensity features of nodular edges, it can reflect the horizontal edge morphology and internal echo situation of nodules in clinical practice. This feature mainly corresponds to the presence of irregularity and lobulation at the edge of the nodule in clinical practice, as well as the high and low echogenicities inside the nodule;
(2) Feature 2 (Wavelet_LH_firstorder-Median): By extracting vertical edge information from transverse ultrasound images and describing the average pixel intensity characteristics of nodular edge images, it can reflect the changes in the vertical edge morphology and internal tissue density of nodules in clinical practice. This feature mainly corresponds to whether the aspect ratio of the nodule is ≥1 in clinical practice, as well as the composition of the nodule;
(3) Feature 3 (Wavelet-LL_firster_10Percentile4G): By extracting the overall structure and low-frequency features of SE ultrasound images, the basal grayscale level of the nodule is described, reflecting the overall shape and basal hardness of the nodule. This feature mainly analyzes the distribution of soft tissue areas in elastic ultrasound images to understand the characteristics of nodules more accurately;
(4) Feature 4 (Wavelet-LH_glszm-SmallArenEmphasis): By extracting the vertical edge textural features of transverse ultrasound images and describing the distribution of high-intensity grayscale areas in nodules, it can reflect the textural complexity and non-uniformity of nodular edges. This feature mainly corresponds to nodular margin lobulation and calcification in clinical practice;
(5) Feature 5 (Wavelet_LL_glcm_lmcl4R): By extracting the overall structure and low-frequency features of strain elastic ultrasound images and describing the frequency of low grayscale pixels appearing in the nodular area, it can reflect the presence of soft tissue or low-hardness areas in the nodule. This feature mainly corresponds to the distribution of the nodular hardness and the contrast between soft and hard areas.

Through the analysis of features, it can be found that for conventional ultrasound, the model mainly focuses on the morphology and characteristics of the edge area of the nodule, corresponding to whether the edge is regular, internally echoes, and calcifies in clinical diagnosis; for USE ultrasound, the model mainly focuses on the overall hardness of the nodules, including the distribution of the nodular hardness and the contrast between soft and hard areas. These results indicate that the proposed model focuses on malignant nodular features that are highly consistent with clinical concerns and have particular clinical significance and credibility.

**Figure 5:**
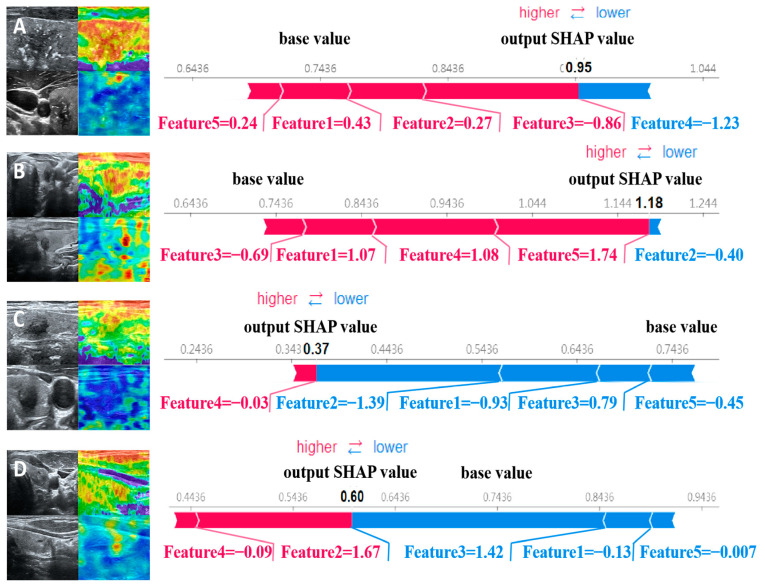
SHAP attempts to explain how our proposed model predicts the benign and malignant thyroid nodules in four patients. Patients (**A**,**B**) have malignant nodules, while patients (**C**,**D**) have benign nodules.

The force plot visually explains the evaluation of individual patients by displaying SHAP values as bar charts, indicating each feature’s positive or negative contributions to the model’s output. Figure 5 shows the predictive probabilities of benign and malignant thyroid nodules for two representative patients. The arrow’s length represents a specific feature’s contribution to the SHAP value (expressed as a percentage). The arrow’s color indicates whether the contribution is positive (red) or negative (blue). Among them, the SHAP values of patients A and B are 0.95 and 1.18, respectively, which are higher than the baseline value of 0.7436, indicating that the model predicts that the patient’s thyroid nodule is malignant; the SHAP values of patients C and D are 0.37 and 0.60, respectively, which are lower than the baseline value of 0.7436. Therefore, the model predicts that the patient’s thyroid nodule is benign.

## 4. Discussion

In this study, we constructed an imaging omics model by fusing multimodal ultrasound image features (including SE, SWE, and corresponding transverse and longitudinal USs) to predict the malignancy of TIRADS 4-5 thyroid nodules. This model can be an easy-to-use and personalized tool to assist clinical diagnosis with high clinical practicality. The ROC curve and various indicators show that our model performs well in diagnostic performance. In the internal testing set and external validation set, the AUC values are 0.85 and 0.79, respectively. In addition, we compare the proposed multimodal feature fusion model with other combined modal models, and the results show that multimodal features have good advantages in predicting the benign and malignant natures of nodules.

Although single modal radiomic models have made significant achievements in the field of diagnosis, they still have shortcomings in identifying complex lesions and small nodules. In contrast, multimodal ultrasound combined diagnosis exhibits higher accuracy [40]. There is relatively little research on multimodal artificial intelligence models based on conventional and elastic ultrasounds [29,30]. Wang et al. constructed a multimodal ultrasound radiomics combined diagnostic model by analyzing conventional ultrasound (US) and elastography (SWE) images [29]. However, the model’s accuracy (ACC) is lower than that of our research results (AUC: 0.829 vs. 0.858). In addition, our study had a larger sample size (508 vs. 130). Zhang et al. developed a predictive model for malignant thyroid nodules by combining radiomic scores from multimodal ultrasound images with independent clinical features through the radiomic analysis of conventional elastography (SE) and shear wave elastography (SWE) images [30]. However, the sample size of this study is relatively small (132 cases) and limited to a single center, and its reliability validation is still insufficient. Therefore, our study constructed an advanced radiomic model by integrating multimodal ultrasound image features. This model has been validated at multiple medical centers and provides a promising, non-invasive, and reliable diagnostic method. This achievement has brought breakthroughs to the field of medical imaging diagnosis. 

Clinical knowledge is crucial in predicting benign and malignant thyroid nodules [41]. Early studies have shown that the shapes (aspect ratios), edges, and compositions of nodules, as well as the internal echogenicity, blood flow, calcification, strain gauge elasticity ratio, and average elasticity value of nodules in ultrasound images are crucial for predicting benign and malignant nodules [42,43]. To integrate these clinical features into the model, we combined the above features with the features extracted using the model. However, the prediction accuracy of the model did not significantly improve. Through analysis, it was found that the essential features selected using the model did not contain any clinical features, which may be because the features extracted using radiomics already contain higher-dimensional clinical information.

In clinical practice, doctors rely on thyroid imaging and their own experience to identify nodules’ benign or malignant nature. However, because of the limitations of image information and the subjectivity of manual judgment, there are risks of misdiagnoses and missed diagnoses in clinical diagnoses. With the latest progress in artificial intelligence, radionic-based models have shown good performance and can become critical auxiliary tools for medical expert decision-making. Although support vector machine (SVM)-based models are widely used and powerful, they can only be applied in clinical practice if the model is explained. To address this issue, it is necessary to develop methods that enable radiologists to understand and trust them. SHAP is a method for interpreting machine-learning models’ predictions, which can decompose complex model predictions to specific numerical contributions of each feature to the prediction, providing a transparent explanation of the model’s predictions. This method helps doctors to understand the model’s decision-making process, enhance their trust in the model’s prediction results, and, thus, more effectively apply it to clinical diagnoses.

This study visually demonstrated the overall impact of various radionic features on the model’s predictions through the SHAP summary plot. First, we found that the five most important features for predicting the malignancy of nodules are all processed through the wavelet transform. The wavelet transform captures more useful features by extracting multiscale and multidirectional information from images, consistent with the clinical approach where doctors need to combine image features from multiple scales and directions for diagnoses. This inspires us to integrate various image features when constructing deep-learning prediction models in the future. Second, through the analysis of features, it can be found that for conventional ultrasound, the model mainly focuses on the morphology and features of the edge area of the nodule. For SE ultrasound, the model mainly focuses on the overall hardness of the nodules. This indicates that the proposed model focuses on malignant nodular features that are highly consistent with clinical concerns and have certain clinical significance and credibility. Finally, the essential features predicted by the model for benign and malignant nodules include information extracted from transverse US, longitudinal US, and SE. However, SWE has almost no effect on the model’s prediction. This may be because the functions of SE and SWE overlap to a certain extent, and the model mainly selects key features from SE.

After clinical doctors understand how features affect SVM models and potential pathophysiological mechanisms, they hope to use the model to evaluate individual outcomes. From the perspective of individual patients, local interpretation of individual patient evaluations can be achieved through the SHAP force plot. Compared with the nomogram method, where clinical doctors calculate certain eigenvalues to generate total scores [44], the SHAP force plot is more time-saving and easier to use. Clinicians can directly compare the output SHAP values of individual patients with the baseline values. In this model, clinical doctors can classify the patient as malignant if the output SHAP value is greater than the baseline value. At the same time, clinical doctors can understand how features affect individual patient evaluations by seeing the arrow’s color (red indicates the power to increase the assessment of the nonresponsive group) and the length of the arrow. The arrow’s length describes a specific feature’s contribution to the evaluation.

In clinical applications, the model proposed in this article can effectively assist clinical doctors in making decisions, especially for small nodules of TI-RADS 4. In this model’s training and testing sets, the range of nodular diameters covers 0.3–3.6cm, which can predict the benign and malignant natures of nodules of most sizes. According to the ACR guidelines, fine-needle aspiration FNA is usually required for TI-RADS 4a nodules with a diameter greater than 1.5 cm and TI-RADS 4b and 4c nodules with a diameter greater than 1.0 cm. Therefore, these larger nodules are still being treated according to ACR guidelines. However, for nodules with a diameter less than 1.5 cm, if the doctor and model agree that they are benign, observation and treatment can be prioritized to reduce unnecessary punctures. When the doctor and model unanimously determine malignancy, it is recommended that the patient undergo a puncture to reduce the risk of a missed diagnosis. In addition, when the judgment results of doctors and models are inconsistent, the key features displayed in the SHAP (Shapley additive explanation) diagram can help doctors to further screen and improve the accuracy of the diagnosis. In summary, the model proposed in this article can enhance doctors’ diagnostic confidence and more effectively persuade patients to accept corresponding treatments, thereby reducing unnecessary punctures and missed diagnoses and optimizing the clinical decision-making process.

This study closely revolves around ultrasound sensor technology. First, we propose an advanced multimodal data fusion method that can effectively integrate data from different modes of the same sensor. This method significantly improves the model’s ability to identify complex lesions and overcomes the limitations of a single ultrasound sensor in terms of contrast and resolution, providing more comprehensive diagnostic information. This method optimizes the model’s performance and provides a new approach for the collaborative application of multiple ultrasound sensor data. Second, the radiomic model proposed in this study has a high degree of universality and can handle data collected using various sensors. This method can effectively refine feature extraction and data fusion for unimodal and multimodal data, thereby improving diagnostic accuracy. At the same time, this method has high scalability and adaptability and can be adjusted according to the characteristics of different sensor data. For example, for sensor data with lower resolution, we can enhance the data quality through specific preprocessing steps to ensure the model’s effectiveness. Finally, the advancement of sensor technology has provided a direct impetus for developing and optimizing this model. With the continuous improvement of sensor resolution, sensitivity, and multimodal fusion capability, the data dimensions and complexity that models can handle have also increased. This bidirectional relationship indicates that the joint development of sensor technology and radiomic models will significantly enhance the overall level of medical imaging diagnosis in the future.

This study significantly improved the accuracy of thyroid nodule diagnosis by innovatively integrating multimodal ultrasound data, such as two-dimensional ultrasound, strain elastography, and transverse wave imaging. We use the LASSO algorithm to screen critical features, related to the malignancy of nodules, from massive ultrasound images, providing an efficient data-processing method for sensors with limited resources. In addition, the model has demonstrated the potential to improve diagnostic performance using machine-learning and deep-learning algorithms, promoting the development of intelligent devices that can automatically adjust parameters to meet different clinical needs and optimize imaging quality. The model exhibits high AUC values in the training and testing sets, particularly in the external validation set, demonstrating its significant advantages in improving diagnostic accuracy and efficiency. This provides direction for improving sensors’ imaging resolutions, sensitivities, and processing speeds. Multidisciplinary collaboration has promoted informational exchange among the fields of biomedicine, electronics, computer science, and clinical medicine, injecting innovative thinking into sensor design and manufacturing. Successful clinical application has validated the model’s effectiveness, accelerated the clinical translation of sensors, and improved the quality and efficiency of medical services. In summary, our multimodal fusion model provides a reliable tool for thyroid nodule diagnosis and lays the foundation for sensor technology innovation and future progress in medical diagnosis.

Our research has some limitations. First, the number of benign nodules in the data is relatively low. Although we reduce this impact by giving higher weights to benign nodules, it may still cause some bias in the model. Future research needs to collect more benign nodule data to balance the training set. Second, this retrospective study requires long-term follow-up and prospective studies to validate the model’s utility. Therefore, in the future, we will include more central data, which may be more helpful in improving the accuracy and robustness of the model. Finally, although we found through SHAP analysis that the features the model focuses on are consistent with the features diagnosed by clinical doctors, this explanation has certain limitations. To maximize the use of AI-assisted diagnosis, it is necessary to establish an appropriate clinical interpretation standard for all radiologists. This will be the focus of our future work.

## 5. Conclusions

Overall, this study utilized data from 468 patients at three medical centers to construct a multimodal deep-learning model for predicting the malignancy of TI-RADS 4 thyroid nodules. The results show that the AUCs of the proposed model reach 0.856 and 0.779 in the testing set and external validation set, respectively, showing excellent diagnostic performance and good generalization ability. More importantly, the method shows a wide range of potential applications in multimodal data fusion and processing and is expected to be extended to other fields of sensor data processing in the future (such as multisource data fusion and improving diagnostic accuracy and efficiency). In addition, using SHAP heatmaps can enable the visual analysis of the impacts of various radiomic features on model predictions, helping radiologists to better understand the model’s decision-making process. In conclusion, the multimodal ultrasound imaging model proposed in this paper performs well and can assist radiologists in diagnosing malignant thyroid nodules more accurately in clinical practice, thereby reducing the risks of unnecessary punctures and missed diagnoses.

## Figures and Tables

**Table 1:** Characteristics of the training, internal testing, and external validation cohorts.

Characteristic	Training Cohort (n = 252)	Testing Cohort	*p*-Value *	Validation Cohort	*p*-Value *
**Final pathology**			0.80		0.02
Benign	78 (25%)	32 (23.9%)		7 (11.3%)	
Malignant	234 (75%)	102 (76.1%)		55 (88.7%)	
**Age (years)**	44.3 [17–77]	45.4 [6–74]	0.34	46 [22–73]	0.29
**Sex**			0.33		0.98
Male	81 (25.9%)	29 (21.6%)		16 (25.8%)	
Female	231 (74.1%)	105 (78.4%)		46 (74.2%)	
**Nodular size (mm)** Maximum diameter	9.02 [2.8–40.4]	8.5 [3–36]	0.94	9.23 [3–31.4]	0.46
**Location**			0.92		0.99
Right	166 (53.2%)	72 (53.7%)		33 (53.2%)	
Left	146 (46.8%)	62 (46.3%)		29 (46.8%)	
**Echogenicity**			0.52		0.83
Hypoechoic	279 (89.4%)	117 (87.3%)		56 (90.3%)	
Isoechogenic or Hyperechoic	33 (10.6%)	17 (12.7%)		6 (9.7%)	
**Microcalcification**			0.54		0.95
Yes	205 (65.7%)	84 (62.7%)		41 (66.1%)	
No	107 (34.3%)	50 (37.3%)		21 (33.9%)	
**Aspect ratio**			0.44		0.01
≥1	132 (42.3%)	62 (46.3%)		37 (59.7%)	
<1	180 (57.7%)	72 (53.7%)		25 (40.3%)	
**Composition**			0.22		0.41
Solid cystic	48 (15.4%)	27 (20.1%)		7 (11.3%)	
Solid	264 (84.6%)	107 (79.9%)		55 (88.7%)	
**Margin**			0.78		0.58
Smooth or ill-defined	187 (59.9%)	85 (63.4%)		33 (53.2%)	
Lobulated or irregular	105 (33.7%)	41 (30.6%)		25 (40.3%)	
Extra-thyroidal extension	20 (6.4%)	8 (6.0%)		4 (6.5%)	
**Blood flow**			0.50		0.52
Yes	224 (71.8%)	92 (68.7%)		47 (75.8%)	
No	88 (28.2%)	42 (31.3%)		15 (24.2%)	
**TI-RADS**			0.19		0.23
4a	205 (65.7%)	95 (70.9%)		35 (56.5%)	
4b	84 (26.9%)	35 (26.1%)		23 (37.1%)	
4c	16 (5.1%)	4 (3.0%)		3 (4.8%)	
5	7 (2.3%) 7	0 (0.0%)		1 (1.6%)	

* Indicates the comparison to the training cohort.

**Table 2:** Evaluated performance results of different modal images and algorithms in training and internal validation sets.

	Training Set			Validation Set		
	ACC	Sensitivity	Specificity	ACC	Sensitivity	Specificity
Combined model	94.6	95.4	92	85.8	87	80.8
Model 1: US	84.3	86.1	78.7	73.1	73.1	73.1
Model 2: US + SWE	83.7	87.3	72	73.1	75	65.4
Model 3: US + SE	94.2	94.9	92	84.3	86.1	76.9

## Data Availability

The data supporting the findings of this study are available on request from the corresponding author (Y.C.).
